# New Remedy for Leucorrhea

**Published:** 1850-05

**Authors:** 


*New Remedy for Leucorrhea.*—In the Boston Medical and Surgical Journal, Dr. Braman states that for nine years he has been in the habit of using preparations of helonias diœcia (unicorn plant) as a remedial agent. He considers it particularly applicable in such affections as have their origin in atony of the generative organs of both sexes—but particularly the female. "In leucorrhea," he says, "I consider it invaluable. I use it with a confidence I attach to no other medicine. Under its influence the patient, whose life has been almost a burden, soon revives. Her uncomfortable sensations vanish, and ultimately, an entire recovery of health and strength is established." It may be given in the form of powder, tincture, or syrup; but the latter is the most eligible.—The doses which Dr. Braman recommends, are of the powder, one drachm and a half; of the tincture, one fluid drachm; of the syrup, three fluid drachms. These doses are to be taken thrice a day, half an hour before the ordinary meals; and according to the urgency of the case, the quantity administered may be increased, if the patient bear it well. In irritable stomachs, nausea is sometimes produced; and when this occurs, the dose must be diminished.

